# Genomic and Transcriptomic Analysis Identified Gene Clusters and Candidate Genes for Oil Content in Peanut (Arachis hypogaea L.)

**DOI:** 10.1007/s11105-018-1088-9

**Published:** 2018-06-23

**Authors:** Xiaohua Wang, Ping Xu, Liang Yin, Yan Ren, Shuangling Li, Yanmao Shi, Thomas D. Alcock, Qing Xiong, Wei Qian, Xiaoyuan Chi, Manish K. Pandey, Rajeev K. Varshney, Mei Yuan

**Affiliations:** 1 Key Laboratory for Peanut Biology, Genetics and Breeding, Ministry of Agriculture, Shandong Peanut Research Institute, Qingdao, 266100 China; 2 Plant and Crop Sciences Division, School of Biosciences, University of Nottingham, Sutton Bonington Campus, Loughborough, LE12 5RD UK; 3 College of Computer and Information Science, Southwest University, Chongqing, 400715 China; 4 College of Agronomy and Biotechnology, Southwest University, Chongqing, 400715 China; 5 International Crops Research Institute for Semi-Arid Tropics (ICRISAT), Hyderabad, 502324 India

**Keywords:** Peanut, Seed oil content, Insertion/deletion (InDel), Genome-wide association study (GWAS), Differentially expressed genes (DEGs)

## Abstract

Peanut (*Arachis hypogaea*), a major source of vegetable oil in many Asian countries, has become an integral part of human diet globally due to its high nutritional properties and option to consume in different forms. In order to meet the demand of vegetable oil, many peanut breeding programs of China have intensified their efforts in increasing oil content in newly bred varieties for reducing the import of edible oils in China. In this context, transcriptome sequencing data generated on 49 peanut cultivars were analyzed to identify candidate genes and develop molecular markers for seed oil content across multiple environments. Transcriptome analysis identified 5458 differentially expressed genes (DEGs) including 2243 positive DEGs and 3215 negative DEGs involved in oil synthesis process. Genome-wide association study identified 48 significant insertion/deletion (InDel) markers associated with seed oil content across five environments. A comparative genomics and transcriptomics analysis detected a total of 147 common gene clusters located in 17 chromosomes. Interestingly, an InDel cluster associated with seed oil content on A03 chromosome was detected in three different environments. Candidate genes identified on A03 form a haplotype, in which variable alleles were found to be different in oil content in an independent population. This locus is important for understanding the genetic control of peanut oil content and may be useful for marker-assisted selection in peanut breeding programs.

**Electronic supplementary material:**

The online version of this article (10.1007/s11105-018-1088-9) contains supplementary material, which is available to authorized users.

## Introduction

Oil crops are the major source of edible oils which are essential part of human food chain and their health. Peanut, also known as groundnut (*Arachis hypogaea* L.; AABB, 2n = 4× = 40), is one of the most important oil crops worldwide, with an average oil content of ~ 50% *w*/*w* (Barrientos-Priego et al. 2002; Yol et al. 2017). Peanut also has been recognized as a functional food due to a well-balanced fatty acid profile and high levels of specific antioxidants and mono- and polyunsaturated fatty acids (Akhtar et al. 2013). Despite the typically high levels of oil content in currently grown peanut varieties, still there is significant variation among germplasm, i.e., ranging from 31.7 to 57.0% *w*/*w* (Yol et al. 2017) which can be used for further enhancing the oil content in newly developed varieties. Because of the above reasons, several breeding programs across the world, including in China, are engaged in developing peanut cultivars with high oil content (Barrientos-Priego et al. 2002; Chen et al. 2016). Despite being such an important trait for several oilseed crops, mere understanding on biological pathway for triacylglycerol (the major form of storage oil in oilseeds) synthesis could be developed (Baud and Lepiniec 2010; Li-Beisson et al. 2013) while the genetic and molecular mechanisms underlying variation in seed oil content of oil crops, including peanut, still remain poorly understood. Improved understanding on genomic control and deployment of genomics tools for several traits successfully accelerated the process and precision in enhancing the genetic gains by developing improved peanut varieties with high trait value (Pandey et al. 2012; Varshney et al. 2013; Pandey et al. 2016; Pandey et al. 2018). Therefore, development and deployment of genomic tools for high oil content will have wider implications in developing improved peanut varieties with enhanced oil content across the globe including China.

Bi-parental populations have been used widely in previous studies to dissect the genetic architecture of complex traits in a number of species followed by discovery of quantitative trait loci (QTLs), candidate genes, and linked markers. Many of these studies have focused on oil content which appears to be a polygenic and quantitative trait due to interactions between multiple genes and the environment. For instance, based on bi-parental linkage mapping, a number of QTL associated with oil content have been identified in all 19 linkage groups of *Brassica napus*, another globally important oil crop (Delourme et al. 2006; Sun et al. 2012; Zhao et al. 2012; Jiang et al. 2014). Further to this, 11 QTLs associated with oil content in soybean have been detected (Eskandari et al. 2013), and 11 QTLs associated with maize kernel oil concentration were detected across nine chromosomes (Yang et al. 2016). In peanut, 6 and 9 QTLs controlling oil content were respectively identified in two different peanut recombinant inbred line (RIL) populations (Pandey et al. 2014a) and 15 QTLs identified in two or more environments were present across environments related to fatty acid concentrations (Wilson et al. 2017). The speedy progress in sequencing technologies reduced the cost of sequencing as well as sequencing-based genotyping which facilitated feasibility of performing genome-wide association studies (GWAS) in addition to alternative to genetic mapping. In contrast to genetic mapping, the GWAS offers a higher-resolution mapping to detect marker-trait associations followed by candidate gene discovery compared with bi-parental linkage mapping due to higher levels of genetic recombination across the population ancestry (Xu et al. 2016). The approach has previously been used successfully to elucidate the control of several important traits in different crops such as drought resistance in *Oryza sativa* (Huang et al. 2010; Courtois et al. 2013), flowering time and leaf architecture in *Zea mays* (Remington et al. 2001; Tian et al. 2011), oil content in *Brassica napus* (Liu et al. 2016), and root system architecture for efficient absorption of phosphorus in *Brassica napus* (Wang et al. 2017).

Cultivated peanut is an allopolyploid species with complex genome structure. There are high levels of homology between the peanut A- and B-subgenome, in which both homologous and non-homologous exchanges have been extensively observed (Bertioli et al. 2016). This complexity imposes a huge challenge for discovery of high-quality molecular markers for use in association studies. So far, the application of association genetics in peanut is rare and only one comprehensive GWAS has been reported for several agronomically important traits (Pandey et al. 2014b). However, several studies have been conducted in other allopolyploid species, indicating the potential application of such techniques in peanut. For instance, Harper et al. (2012) identified genetic loci controlling glucosinolate and erucic acid content using association analyses based on transcriptome data in a population of 53 *Brassica napus* (*Brassica* AACC; 2n = 38) genotypes. The use of transcriptome data reduced the complexity typically associated with polyploid genomes, as well as allowing the use of both single nucleotide polymorphism (SNP) markers and gene expression markers (GEMs) by measuring transcript abundance. Further studies using the same techniques in larger populations of 84 and 101 genotypes identified genetic loci associated with the control of anion homeostasis and seed glucosinolate concentration (Koprivova et al. 2014; Lu et al. 2014). Another association study deployed *Brassica* 60K SNP array on a panel of 472 rapeseed genotypes and identified 24,256 polymorphic SNPs which facilitated detection of a region on chromosome A08 significantly associated with seed oil content (Li et al. 2014). The above examples indicate the ability of association genetics to identify loci controlling such complex traits such as oil content in polyploid organisms.

After SNPs, insertions and deletions (InDels), originated from replication slippage, transposable elements, and crossing-over, are the second most abundant structural variations in plant genomes including peanut. These markers have an array of applications including genetic diversity, trait mapping, and molecular breeding. Therefore, in the present study, we developed and deployed a total of 61,942 insertion–deletion (InDel) markers from the transcriptome of peanut pods for conducting GWAS to identify genetic loci associated with oil content. The genomic loci and candidate genes identified in this study have improved understanding on genomic control for high oil content in addition to possibility of developing user-friendly genetic markers for use in molecular breeding for enhancing the level of oil content in improved peanut varieties.

## Materials and Methods

### Plant Materials

The association panel comprised of 49 peanut accessions which were collected from major breeding centers located in China, i.e., 28 lines from Shandong; 5 from Henan; 4 from Sichuan; 3 from Guangdong; 2 from Hubei;1 each from Hebei, Fujian, and Shanxi; 3 from the USA; and 1 from Mali (Supplementary Table 1).

### Field Experiments and Phenotyping

All the 49 genotypes were planted at Laixi, China (120.53° E, 36.86° N), in 2015–2016 and at Qingdao, China (120.41° E, 36.39° N), in 2014–2015 and 2015–2016 and in greenhouse in 2015–2016 at Qingdao. All the field experiments followed a randomized block design with three replicates. Twelve individual plants were planted in a single row for each accession. The trial management followed standard breeding field protocols. At maturity stage, five representative plants from the center of each plot were harvested. The oil content of the desiccated seeds was determined by Soxhlet extraction (ISO 659:^2009^), and the “oil content” *w*, expressed as percentage by mass of the product as received, is given by the following:

$$ w={w}_1-\left[\frac{p+{I}_0+{I}_n}{100}\right]\left({w}_1-{w}_2\right), $$

where

*p*: is the percentage, by mass, of total fines;
*I*_0_: is the percentage, by mass, of oleaginous impurities;
*I*_*n*_: is the percentage, by mass, of non-oleaginous impurities;
*w*_1_: is the percentage, by mass, of oil in the pure seeds; and
*w*_2_: is the percentage, by mass, of oil in the impurities.

### Calculation of the Best Linear Unbiased Prediction of Oil Content

As the oil content of the peanut association panel was investigated in multiple environments at multiple time points with multiple replications, the best linear unbiased prediction (BLUP) of oil content for each line across the four environments was calculated using an R script (www.eXtension.org/pages/61006, 20 November 2015, date last accessed) based on a linear model. Means (BLUP) were estimated using the (genotype) term as a fixed factor, retaining [(replicate/row/location/year)] as random factors. Random terms and no defined fixed factor were used to estimate sources of variation. These data were handled separately in an additional GWAS.

### Genotyping of the Association Panel and In Silico Mapping of Markers

The 30–40 days immature pods (stages 2–3) from each of the 49 accessions were sampled as explained in Lynch and Wilson (1991). Samples were cleaned and immediately placed in liquid nitrogen before being stored at − 80 °C (Wei et al. 2016). The sequencing libraries of 147 RNA samples were generated using the Illumina RNA Library Prep Kit (NEB #E7760, San Diego, CA, USA) and sequenced on an Illumina Hiseq 2000 platform with 100-bp paired-end reads. Sequencing reads were aligned to the peanut “pseudomolecules” reference genome (AA, *Arachis duranensis*; BB, *Arachis ipaensis*, https://www.peanutbase.org/home) with Bowtie2 (Langmead et al. 2012) and then assembled using TopHat 2.0.0 and Cufflinks (Trapnell et al. 2012).

The InDel markers detected by GATK software (https://gatkforums.broadinstitute.org/gatk/) with call frequencies missing < 20% and minor allele frequencies (MAF) > 0.05 were selected for association mapping analysis. The physical position of the markers was identified by aligning the sequence of a 100-bp paired-end reads attached to each marker with the “pseudomolecules” genome sequences of peanut (*Arachis duranensis*—AA and *Arachis ipaensis*—BB) using local BLASTn (BLAST, Basic Local Alignment Search Tool; http://blast.ncbi.nlm.nih.gov/Blast.cgi). If the reads matched two or more locations in the reference genome of peanut, the markers were regarded as non-specific markers and discarded.

### Population Structure and Relative Kinship Analysis of the Association Panel

Genotype–phenotype covariance can lead to spurious associations in GWAS due to unknown ancestry (Lander and Schork 1994). Population structure and kinship can be estimated and used in linear models to correct for such false associations. Using the Bayesian Markov chain Monte Carlo (MCMC) model in STRUCTURE V. 2.3.4 (Xu et al. 2016; Wang et al. 2017), the population structure of the peanut association panel was estimated. Based on a model for admixture and correlated allele frequencies in STRUCTURE V.2.3.4, a population parameter (*K*) value was obtained across five iterative runs with a putative number of populations set from 1 to 10. The length of the burn-in period and number of MCMC replications after burn-in were each set to 100,000. After calculating the rate of change in log probability of the data (LnP(D)) and an ad hoc statistic Δ*K* between successive *K* values, the true *K* value was determined (Evanno et al. 2005). A relative kinship matrix was calculated by SPAGeDi (http://ebe.ulb.ac.be/ebe/SPAGeDi.html) with all negative kinship values between two individuals set to 0 (Wang et al. 2017).

### Genome-Wide Association Analysis of Oil Content Traits

Population structure and kinship could be used for correcting the spurious association created by genotype–phenotype covariance. Generalized linear model (GLM) and mixed linear model (MLM) calculations were performed in TASSEL version 5.0 to determine the best model for association analyses. The GLM included a naïve model without controlling for population structure and a Q model which controlled for population structure using a Q matrix to identify subpopulation fit as covariate in the general linear model. The MLM included a K model which assesses pair-wise relationship between the individuals using the kinship matrix obtained in SPAGeDi software and a Q+K model which controlled for population structure using subpopulation and kinship between the genotypes. Quantile–quantile plots were created with a negative log_10_(*P*) value of the expected *P* value from the genotype–phenotype association and the expected *P* value under the assumption that no association exists between genotype and phenotype. The threshold of significance was set to *P* < 1.61 × 10^−5^ (1/total markers used) (Wei et al. 2016). The phenotype or trait was determined by GWAS, and the trait *y* is given by the following (Yu et al. 2006):

$$ y=\mathrm{X}\alpha +\mathrm{S}\beta +\mathrm{Q}\sigma +\mathrm{Z}\delta +e, $$

where

*y*: is the trait values,
*α*: is the environments,
*σ*: is the candidate markers effects,
*γ*: is the subpopulation effects, and
*δ*: is the background genetic effects.

### Differentially Expressed Genes Underlying Significant SNP Loci and Favorable Alleles for Seed Oil Content

Three high oil content cultivars and three low oil content cultivars selected from the panel of 49 cultivars for differentially expressed gene (DEG) analysis. Differentially expressed genes between high and low were identified based on criteria FDR < 0.01 and |log_2_ (FPKM_high_)/FPKM_low_| > 2. Based on physical map and gene annotation, the genes located in the LD region of the significant InDel markers “InDel6418” were identified at three different environments (Oilcontent2016-QD2, Oilcontent2016-QD1, and OilContent-BLUP). Due to an inconsistent number of SNPs for each InDel marker, SNP markers in a locus associated with peanut oil content on chromosome were used to analyze the relationship between markers haplotypes and phenotypes in the association panel. More than three cultivars with the same SNP defined a haplotype. The cultivar phenotypes were figured by Origin 8 software.

## Results

### Phenotypic Variation of Peanut Seed Oil Content

Extensive phenotypic variation was observed in oil content within and across different environments. Across the five environments, the seed oil content of the peanut association panel showed continuous variation and approximated a normal distribution (Supplementary Fig. 1). Peanut seed oil content ranged from 40.01 to 58.11%, with an average of 48.10% in 2016 at Qingdao2 environment; from 44.35 to 59.34%, with an average of 52.74% in 2016 at Qingdao1 environment; from 43.66 to 57.86%, with an average of 50.41% in 2016 at Laixi environment; and from 46.40 to 58.00%, with an average of 50.60% in 2015 at Qingdao environment (Table 1). The oil content coefficient of variation ranged from 5.37 to 9.47%, with an average of 6.93%, suggesting that the oil content of most genotypes is stable in different environments and relatively insensitive to environmental factors.

**Table 1:** Mean, standard deviation (SD), maximum (Max), minimum (Min), and coefficient of variation (%) of the seed oil content of peanut in five different environments and the best linear unbiased prediction of oil content

Traits	Mean ± SD	Max	Min	CV (%)
OilContent 2015-Qingdao	50.60 ± 2.72	58.00	46.40	5.37
OilContent 2016-Laixi	50.45 ± 3.31	57.86	43.66	6.55
OilContent 2016-Qingdao1	48.10 ± 4.55	58.11	40.01	9.47
OilContent 2016-Qingdao2	52.74 ± 3.82	59.34	44.35	7.25
OilContent-BLUP	50.47 ± 3.03	56.59	44.49	6.01

### Population Structure and Relative Kinship

A total of 61,942 InDels with MAF > 0.05 and call frequencies > 0.8 were matched to a unique position in the “pseudomolecules” of diploid genome sequences of peanut progenitors (*Arachis duranensis*—AA and *Arachis ipaensis*—BB) (Supplementary Table 2). These InDels were used to assess the population structure and relative kinship of the association panel, in addition to association analyses. A total of 61,942 InDel markers were identified across all chromosomes. InDel number on each chromosome ranged from 2069 on A07 to 4724 on B03. The density of InDel markers on each chromosome ranged from 46.74 kb/InDel on B08 to 17.96 kb/InDel on A08 (Table 2).

**Table 2:** The number and density of InDel markers detected across peanut chromosomes

Chromosome	InDel number	Reference length (Mb)	Density of markers (kb/InDel)
A01	2615	106.00	40.54
A02	2370	92.60	39.07
A03	4200	133.13	31.70
A04	2632	121.18	46.04
A05	3243	108.28	33.39
A06	3025	110.73	36.60
A07	2069	77.95	37.68
A08	2725	48.94	17.96
A09	2866	119.00	41.52
A10	2475	107.25	43.33
B01	3052	136.90	44.86
B02	2893	108.60	37.54
B03	4724	135.60	28.70
B04	3123	133.20	42.65
B05	3904	149.40	38.27
B06	3518	136.70	38.86
B07	3050	126.00	41.31
B08	2762	129.10	46.74
B09	3442	146.50	42.56
B10	3254	135.80	41.73

**Fig. 1:**
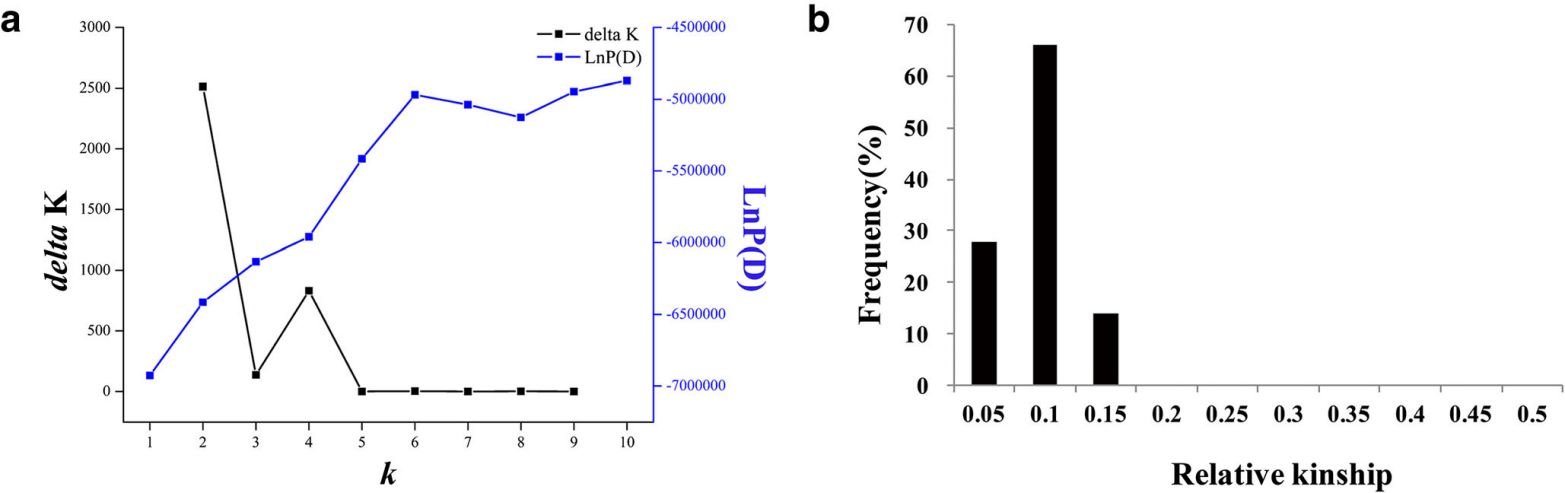
The population structure and relative kinship of the peanut panel. **a** The rate of change in log probability of the data (LnP(D)) and ad hoc statistic Δ*K* (delta *K*) of population structure in the 49 peanut population association panel. **b** The relative kinship of the peanut panel

The *K* values obtained through population structure in the peanut association panel increased continuously with no obvious inflection points of LnP(*K*) values (Fig. 1a). The *∆K* values indicated that the rate of change ad hoc statistic was equal to 2; therefore, the population could be divided into two subgroups: subgroup 1 and subgroup 2 (Supplementary Table 1). Of the four lines grouped together in subgroup 1, three originated from Shandong while single lines were from Guangzhou. Subgroup 2 was big with 45 lines originated from Shandong, Fujian, Guangdong, Hebei, Henan, Hubei, Shanxi, Sichuan, the USA, and Mali (Supplementary Table 1). The relative kinship between genotypes in the association panel analysis showed that the genotypes with kinship coefficients less than 0.1 accounted for 79.77% of the association panel (Fig. 1b), indicating that the majority of the genotypes have a weak relationship with one another.

### Genome-Wide Association Analysis and Gene Expression Profile of Candidate Genes

**Fig. 2:**
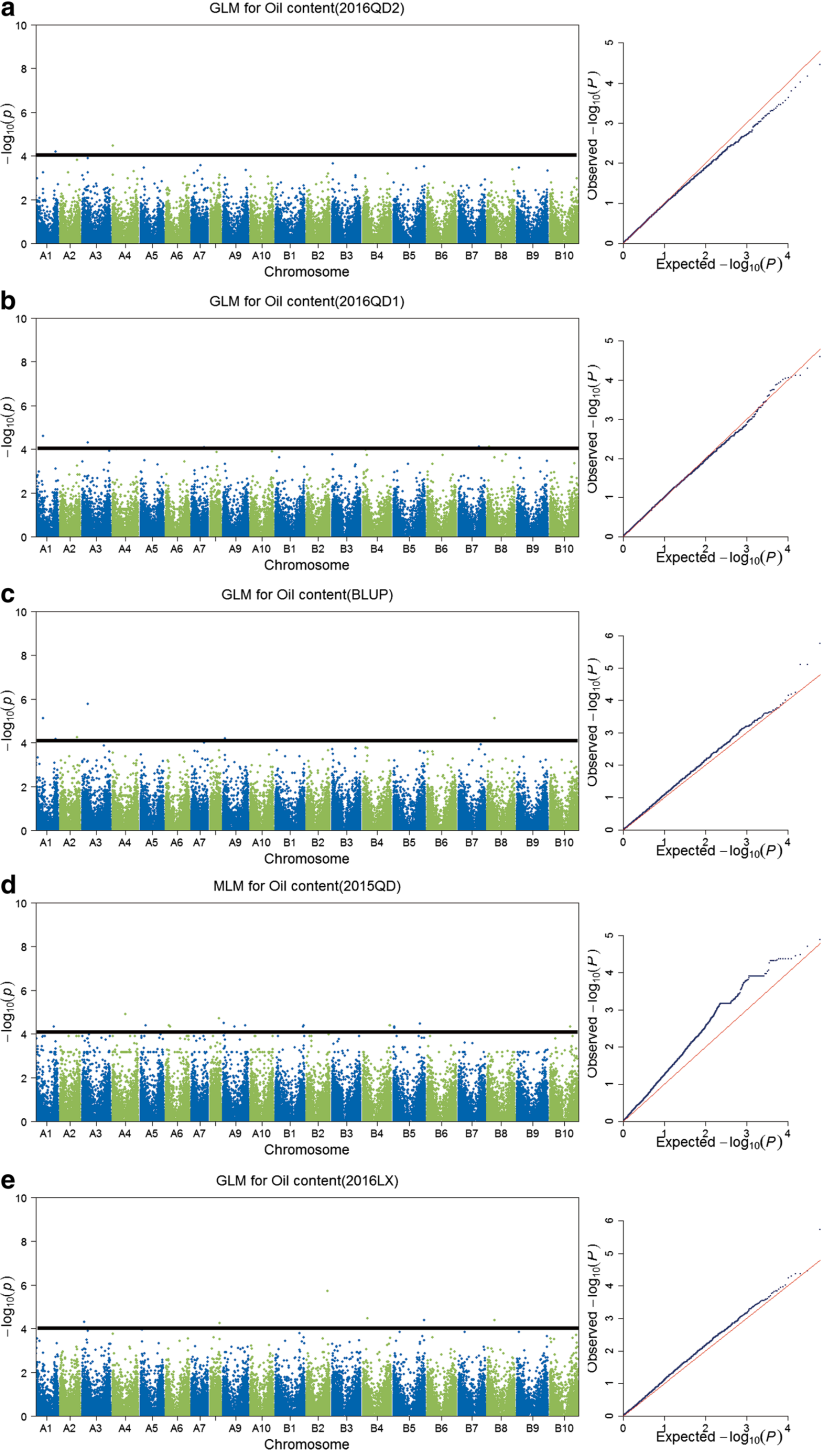
Genome-wide association scanned for oil content of peanut. **a** Manhattan and optimal quantile–quantile plot for oil content detected in 2016QD2 environment. **b** Manhattan and optimal quantile–quantile plot for oil content detected in 2016QD1 environment. **c** Manhattan and optimal quantile–quantile plot for oil content using the BLUP value. **d** Manhattan and optimal quantile–quantile plot for oil content detected in 2015QD environment. **e** Manhattan and optimal quantile–quantile plot for oil content detected in 2016LX environment

**Fig. 3:**
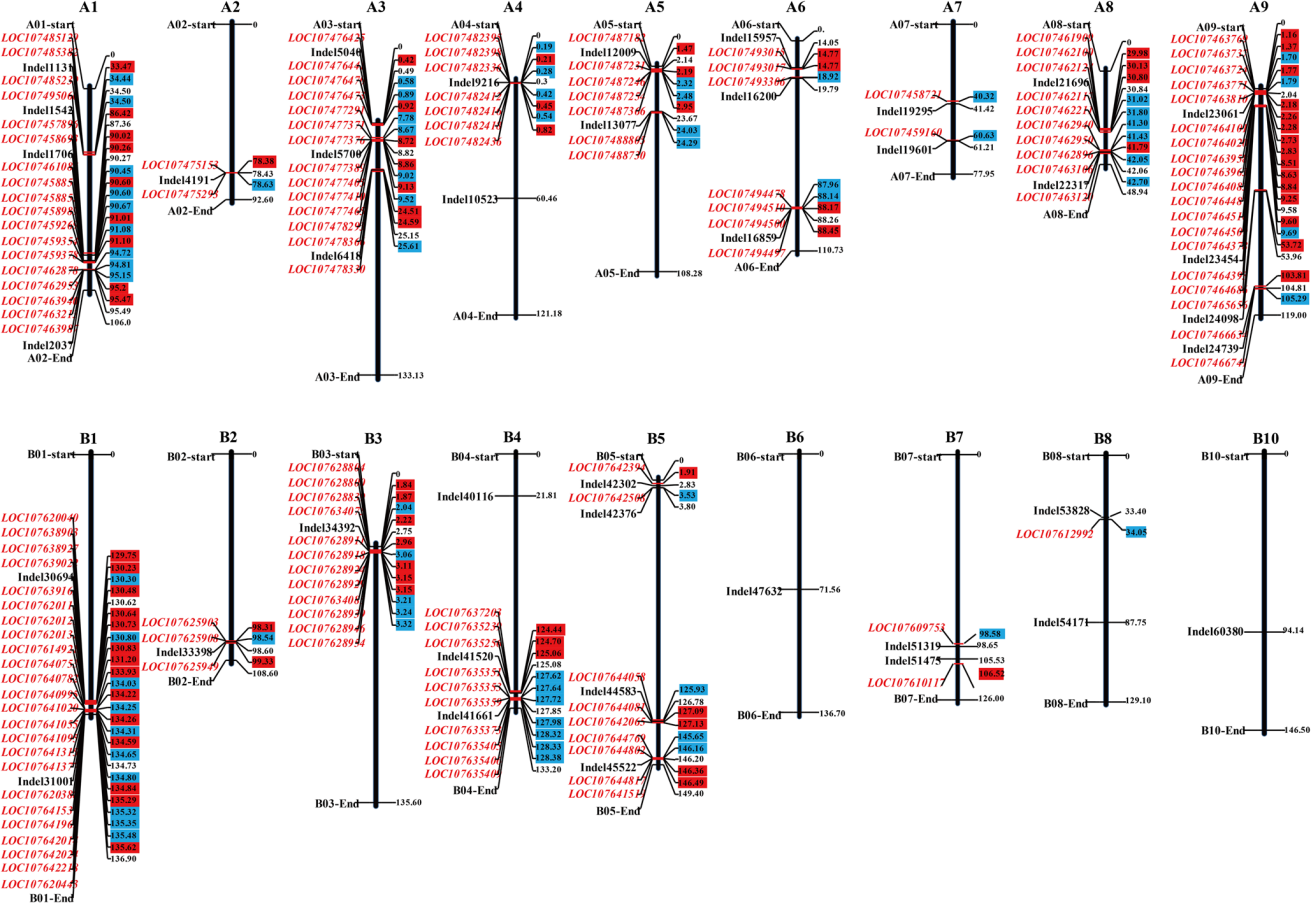
The distribution pattern of 147 candidate differential expression genes (DEGs) and their corresponding InDels associated with oil content. InDels and candidate genes are marked in black and red, respectively. Positive and negative DEGs are marked in red blocks and blue blocks

**Fig. 4:**
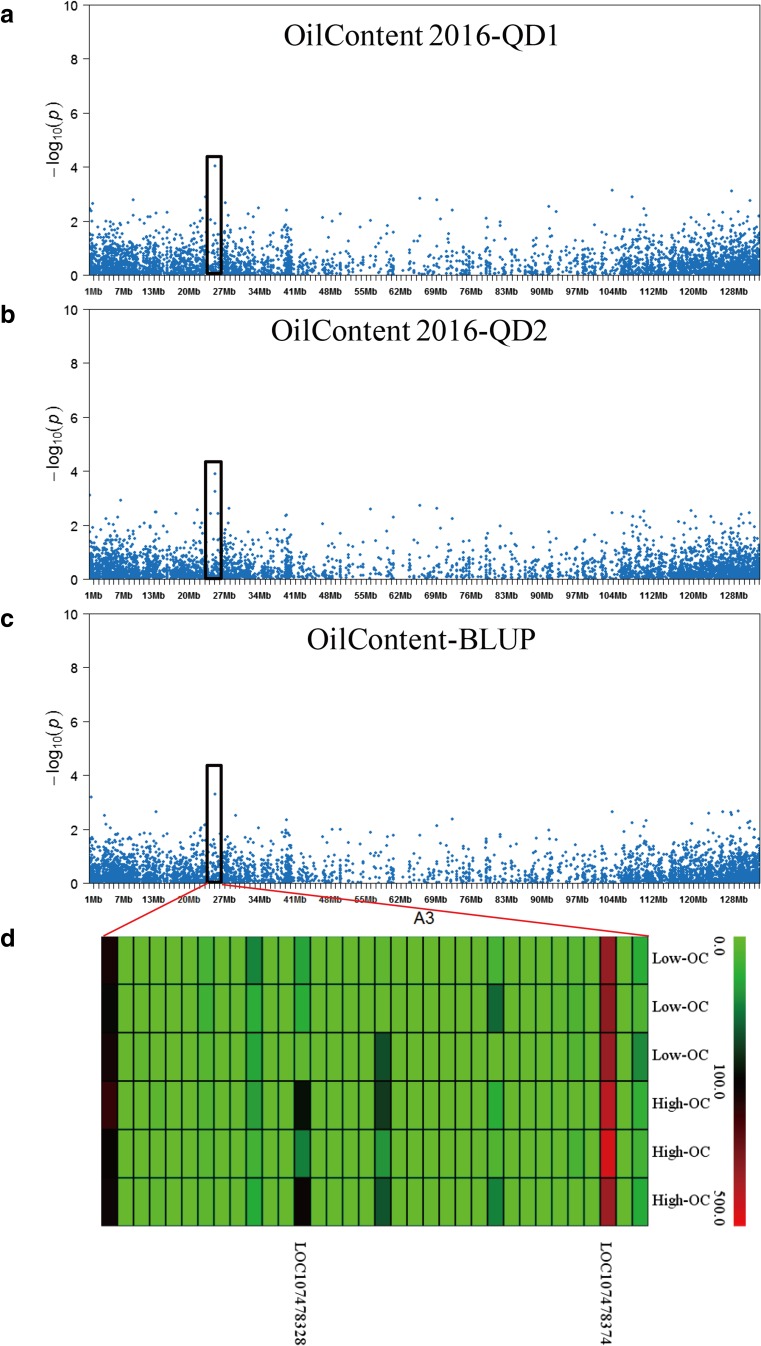
Co-localized loci on chromosome A03 for oil content of peanut. **a** Significant InDel associated with oil content in 2016QD1 environment. **b** Significant InDel associated with oil content in 2016QD2 environment. **c** Significant InDel associated with oil content using the BLUP value. **d** Expression profile detected by transcriptome sequencing of candidate genes located in the LD intervals

The Naïve model, Q model, K model, and Q+K model were used for association mapping. The deviations of observed values from the expected values are shown in QQ plots (Supplementary Fig. 2) and were applied to select the most suitable model for each trait in the different experiments (Fig. 2a–e). Based on the results of QQ plot, Q+K, K, K, GLM, and K models are fitful for 2015Qingdao, 2016Laixi, 2016Qingdao1, 2016Qingdao2, and mean (BLUP) environments, respectively. A total number of 19, 5, 10, 8, and 6 significant InDel markers for oil content were detected in 2015Qingdao, 2016Laixi, 2016Qingdao1, 2016Qingdao2 and mean (BLUP) environments, respectively (Fig. 2; Supplementary Table 3). The GWAS analysis identified a total of 48 significant loci for oil content in five different environments. The significant loci were located across 18 chromosomes (Fig. 3; Supplementary Table 3). One of the significant loci (InDel6418) located on A03 chromosome was detected across three different environments (Fig. 4). This locus had shown high phenotypic variation explained (PVE, nearly 30%) for oil content indicating its importance for further investigation on its role in oil biosynthesis mechanism and use in molecular breeding.

After ranking the genotypes in the association panel by oil content (Supplementary Fig. 3), three high (31, 44, 46) and three low (7, 8, 14) oil content genotypes were selected for expression profiling of candidate genes related to oil content (Supplementary Table 4). A total of 5458 differently expressed genes were identified between high and low oil content genotypes (Supplementary Table 5). A comparison of our GWAS and transcriptome sequencing results revealed 147 common gene clusters located in 17 chromosomes in peanut (Supplementary Fig. 3). Genes at these loci may be involved in the control of peanut oil content and are worthy for further investigation.

### Haplotypes Associated with Peanut Oil Content Traits

**Fig. 5:**
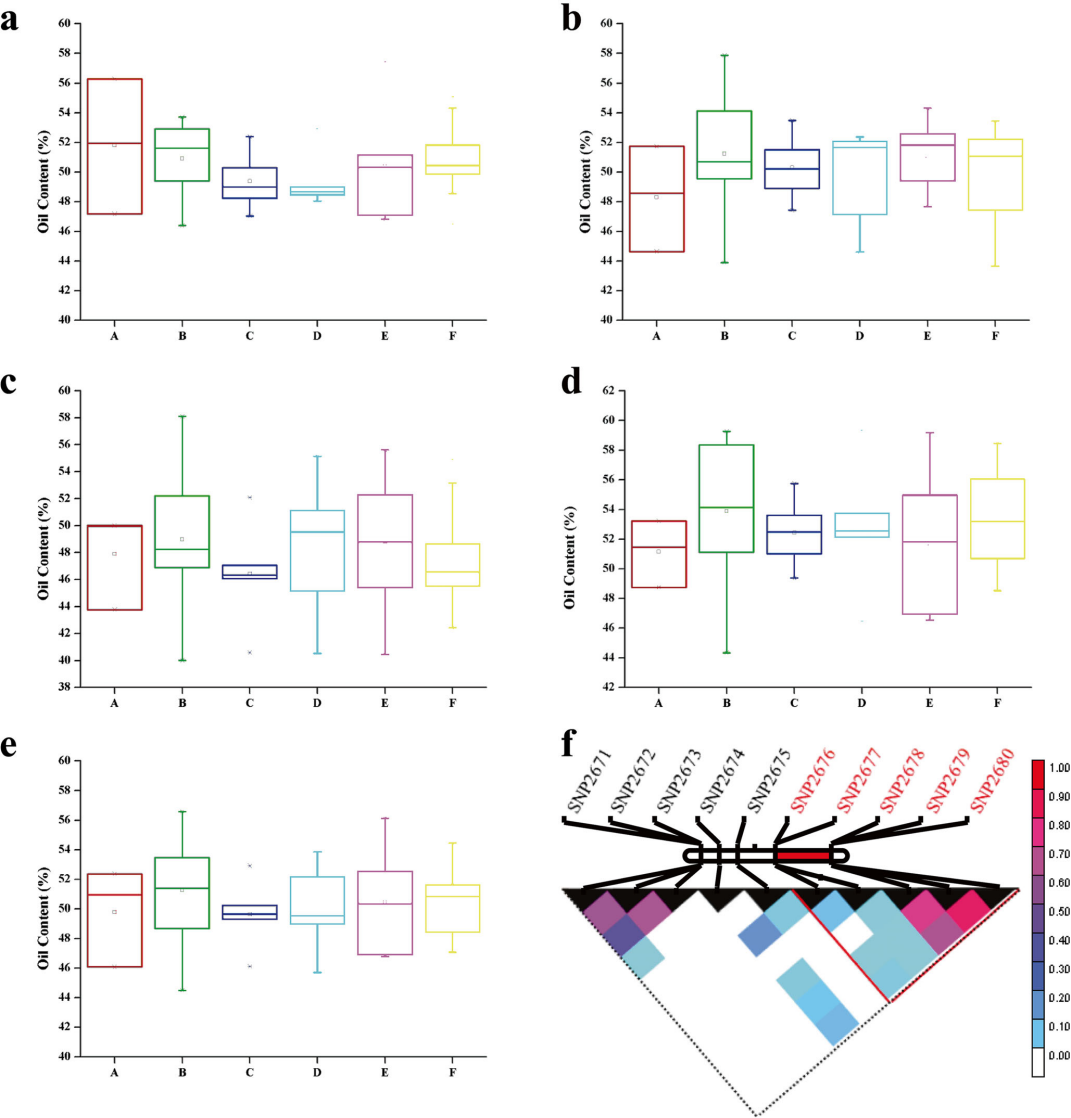
The haplotypes associated with oil content traits in the five different environments. **a** The haplotypes associated with oil content in 2015QD environment. **b** Oil content in 2016QD1 environment. **c** Oil content in 2016QD2 environment. **d** Oil content in 2016LX environment. **e** Oil content using the BLUP value. **f** The haplotypes on A03 (red color trilateral)

On chromosome A03, 34 genes were housed in the candidate genomic interval which is home for the significant InDel6418 (CTTTTTT/-) locus (Fig. 4d) detected at three different environments (Oilcontent2016-QD2, Oilcontent2016-QD1, and OilContent-BLUP). Two genes (*LOC107478374* and *LC10747828*) were located in the interval region of the peak marker InDel6418 at 24.15–26.15 Mb. There were ten SNPs located in this interval (Fig. 5f). Five of ten SNPs formed a region with five different haplotypes on A03 chromosome. The cultivars or inbred lines with haplotype 2 (CTTGA, *n* = 13) had higher average peanut oil content (51.26 ± 4.44%) than those with other haplotypes in five environments (Fig. 5a–e), whereas the varieties with haplotype 3 (YTTGA, *n* = 5) had lower average peanut oil content (49.64 ± 3.20%) than those with the other haplotypes (Fig. 5a–e).

## Discussion

Development of peanut varieties with high oil content is the most important breeding goals after pod yield for peanut improvement programs globally. Detection of genetic loci related to oil content could guide such breeding efforts in more fast and efficient manner. Previous studies based on bi-parental linkage mapping for seed oil content have identified ten main-effect QTLs for oil content and oil quality traits of peanut (Pandey et al. 2014b). Fifteen QTLs identified in two or more environments that were present across environments related to fatty acid concentrations were detected in peanut BC_3_F_6_ population (Wilson et al. 2017). GWAS performed with a large number of SNPs and InDels has been reported in many plants and crops, such as *Arabidopsis* (Atwell et al. 2010), rice (Huang et al. 2010), and maize (Kump et al. 2011; Li et al. 2013). There were many oil content studies reported in recent years where single locus (*BnaA.FAE*) to 50 loci significantly associated with seed oil content were identified in *B. napus* (Li et al. 2014; Liu et al. 2016). In this study, 48 loci significantly associated with peanut oil content were detected across multiple environments. A total of 6 of 48 significant loci were located in more than two different environments (Supplementary Table 3).

There are many elements that affect the power of GWAS such as marker density, population size, and statistical methods. Markers can be obtained by the advances in genome sequencing such as reference genome sequences of diploid progenitors of cultivated tetraploid (Bertioli et al. 2016) and 58K SNP arrays (Pandey et al. 2017) in peanut. In the present study, we have successfully identified > 60K InDels and 20K SNP markers through mRNA-seq (Supplementary Table 2; Supplementary Table 4). Population structure and relative kinship were used for controlling false-positive results in GWAS. The association panel was classified into two subpopulations (Fig. 1a, c) in our studies, co-incidentally similar to studies conducted with elite varieties in rice (Huang et al. 2010) and rapeseed (Xu et al. 2016; Liu et al. 2016; Wang et al. 2017). The *K* matrix (unequal relatedness among individuals) was the main elements of MLM statistical models. In the present study, genotypes with kinship coefficients less than 0.1 accounted for 79.77% of the association panel (Fig. 1b), indicating that the genotypes in the association panel were only weakly related. Four different models were constructed for controlling spurious associations, such as the naïve model, Q model, K model, and Q+K model (Supplementary Fig. 2). When kinship is ignored in our association study, the *P* values were found to be significantly inflated, indicating that relative kinship within the population is likely to be one of the major factors resulting in false positives (Supplementary Fig. 2). With the addition of kinship in the models, such false positives are controlled in the results obtained in the present investigation.

Oil content is one of most important agricultural traits in peanut. Previous studies on the high oil content of peanut were mainly conducted using QTL mapping (Pandey et al. 2014b). However, GWAS were implemented in a collection of peanut germplasms in our studies (Supplementary Table 1). Oil content is a complex trait and our investigation identified 5458 DEGs including 2243 positive DEGs and 3215 negative DEGs involved in the oil synthesis process (Fig. 3; Supplementary Table 5). Breeders have matched functionally different oil-related alleles to specific environments during the peanut breeding process (Liu et al. 2016). In this study, the association panel of 49 genotypes of peanut was planted in five different environments and the seed oil content quantified (Supplementary Fig. 3). Five SNPs near a significant InDel marker on chromosome A03 formed a haplotype which was shown to contribute a major percentage of peanut oil content (Fig. 5). Favorable alleles at this locus may be an effective way to increase the oil content during the selection of high oil lines using molecular breeding. The discovery of an optimal haplotype on A03 provides a useful resource which may enable accurate selection of peanut with higher seed oil content and improve our understanding of the molecular mechanisms related to oil accumulation in plants.

## Electronic Supplementary Material

ESM 1 (XLSX 12 kb)

ESM 2 (XLSX 14354 kb)

ESM 3 (XLSX 12 kb)

ESM 4 (XLSX 24388 kb)

ESM 5 (XLSX 581 kb)

ESM 6 (DOC 280 kb)

ESM 7 (DOC 131 kb)

ESM 8 (DOC 87 kb)
