# Supplementary material for: Genomic and Transcriptomic Analysis Identified Gene Clusters and Candidate Genes for Oil Content in Peanut (Arachis hypogaea L.)
Source: Plant Mol Biol Report. 2018 Jun 23;36(3):518–29. doi: 10.1007/s11105-018-1088-9 (PMC6061501; doi:10.1007/s11105-018-1088-9)
Supplement: Supplementary file 8 — (DOC 87 kb) [file 11105_2018_1088_MOESM8_ESM.doc]

**Genetic variants associated analysis and differential expression analysis of oil content in peanut (*Arachis hypogaea* L.)**

XiaohuaWang^1^**^†^**, Ping Xu^1^**^†^**, Liang Yin^1^**^†^**， Yan Ren^1^, Shuangling Li^1^, Yanmao Shi ^1^, Thomas D Alcock^2^, Qing Xiong^3^，Wei Qian^3^，Xiaoyuan Chi^1^, Manish K Pandey^4^, Rajeev K Varshey^4^, Mei Yuan^1*^

**Supplementary Figure 3.** The high and low oil content cultivars were selected in panel in five different environments. High and low oil content cultivar were marked in red and green columns, respectively.
